# Integrated care, financing and multimorbidity. How to bridge the gaps?

**Published:** 2012-09-04

**Authors:** Jeroen Struijs, S.R de Bruin, C.A Baan

**Affiliations:** Senior Researcher RIVM, National Institute for Public Health and the Environment, Centre for Prevention and Health Services Research, Bilthoven, The Netherlands; National Institute for Public Health and the Environment, Centre for Prevention and Health Services Research, Bilthoven, The Netherlands; National Institute for Public Health and the Environment, Centre for Prevention and Health Services Research, Bilthoven, The Netherlands

**Keywords:** Integrated care, financing, multimorbidity, bundled payment

## Abstract

In many Western countries, including the Netherlands, disease management programs are increasingly being implemented to enhance quality and continuity of care for patients with chronic conditions. Fragmentary funding of these programs, however, hampered the establishment of long-term programs. In 2007, the Dutch minister of health therefore approved the introduction of a bundled-payment approach to stimulate the implementation of disease management. This was initially on an experimental basis for single-disease programs including programs for diabetes patients. In 2010, the bundled-payment concept was approved for nationwide implementation for diabetes, COPD and vascular risk management.

Under this system, insurers pay a single fee to cover a full range of chronic care services for a fixed period. One of the key questions regarding this bundled payment approach is how to deal with patients with multimorbidity. Participation of these patients in multiple single-disease oriented programs may again lead to fragmented care.

The workshop comprises three presentations. The first presentation will provide an overview of disease management initiatives and evidence for their effects on quality of care and health care costs. During this presentation also payment reforms regarding disease management, among which pay-for-performance and bundled payments, will be discussed.

During the second presentation the basic premises of the Dutch bundled payment model for diabetes care will discussed in-depth. Further, the results of a three-year follow-up study evaluating the experiences with bundled payment will be presented. Data from electronic health records, extensive interviews with stakeholders, and patient questionnaires distributed in a random sample of diabetic patients were used to assess the satisfaction of all stakeholders and the quality of delivered care. The third presentation focuses on care programs for patients with multimorbidity. During this presentation the results of a systematic review on care programs for this target group will be summarized. After the presentations a discussion will be held on how to bridge the gap between multimorbidity and the single-disease management approaches funded by bundled payments.

